# Thiazolidinediones and Edema: Recent Advances in the Pathogenesis of Thiazolidinediones-Induced Renal Sodium Retention

**DOI:** 10.1155/2015/646423

**Published:** 2015-05-14

**Authors:** Shoko Horita, Motonobu Nakamura, Nobuhiko Satoh, Masashi Suzuki, George Seki

**Affiliations:** Department of Internal Medicine, The University of Tokyo Hospital, Tokyo 113-0033, Japan

## Abstract

Thiazolidinediones (TZDs) are one of the major classes of antidiabetic drugs that are used widely. TZDs improve insulin resistance by activating peroxisome proliferator-activated receptor gamma (PPAR*γ*) and ameliorate diabetic and other nephropathies, at least, in experimental animals. However, TZDs have side effects, such as edema, congestive heart failure, and bone fracture, and may increase bladder cancer risk. Edema and heart failure, which both probably originate from renal sodium retention, are of great importance because these side effects make it difficult to continue the use of TZDs. However, the pathogenesis of edema remains a matter of controversy. Initially, upregulation of the epithelial sodium channel (ENaC) in the collecting ducts by TZDs was thought to be the primary cause of edema. However, the results of other studies do not support this view. Recent data suggest the involvement of transporters in the proximal tubule, such as sodium-bicarbonate cotransporter and sodium-proton exchanger. Other studies have suggested that sodium-potassium-chloride cotransporter 2 in the thick ascending limb of Henle and aquaporins are also possible targets for TZDs. This paper will discuss the recent advances in the pathogenesis of TZD-induced sodium reabsorption in the renal tubules and edema.

## 1. The Target of Thiazolidinediones: Peroxisome Proliferator-Activated Receptor Gamma (PPAR*γ*)

Peroxisome proliferator-activated receptors (PPARs) belong to the nuclear receptor superfamily of ligand-inducible transcription factors [1], involved in lipid metabolism and energy homeostasis [2]. In mammals, three PPAR subtypes, PPAR*α*, PPAR*β*/*δ*, and PPAR*γ*, are known to exist. PPARs bind to PPAR-responsive regulatory elements (PRREs) in combination with retinoid X receptor (RXR) and control the expression of genes engaged in several biological processes, such as lipid metabolism, adipogenesis, inflammation, and maintenance of metabolic homeostasis [3]. PPARs consist of an N-terminal transactivation domain, which is quite diverse and contains the AF1, a DNA-binding domain (DBD), which is highly conserved, and a ligand-binding domain (LBD) in the C-terminal, which contains the AF2 [4].

PPAR*γ* has two isoforms, PPAR*γ*1 and PPAR*γ*2 [5, 6]. PPAR*γ*2 is longer than PPAR*γ*1, with an extra 30 amino acids at its N-terminus. PPAR*γ*1 is expressed in a wide range of tissue types, which include white and brown adipose tissue, cardiac muscle, and liver tissue, whereas PPAR*γ*2 is expressed almost exclusively in adipose tissue [7, 8]. However, the expression of PPAR*γ*2 is induced in other tissues by a high fat diet [9].

PPAR*γ* is a key regulator of adipogenesis [1, 10]. It is expressed abundantly in white and brown adipocytes and plays important roles in regulating lipid metabolism and insulin sensitivity. Additionally, PPAR*γ* functions as a multipotent modulator of inflammation, gluconeogenesis, and fluid homeostasis.

Disruption of the PPAR*γ* gene in mice yielded intriguing results [11]. Homozygous PPAR*γ* deficiency led to embryonic lethality due to placental dysfunction. Embryonic fibroblasts from PPAR*γ* ^−/−^ mice failed to differentiate into adipocytes, suggesting that PPAR*γ* is essential for the differentiation of embryonic fibroblasts into adipocytes. On the other hand, heterozygous PPAR*γ* ^+/−^ mice gained little weight under a high-fat diet. Moreover, the PPAR*γ* ^+/−^ mice had higher sensitivity to endogenous insulin than wild-type mice. PPAR*γ* may have dual roles in regulating insulin resistance, at least in experimental mice.

## 2. PPAR*γ* and Kidney

In the kidney, PPAR*γ* is mainly expressed in the collecting ducts. However, some studies have shown that PPAR*γ* is also expressed in other nephron segments, such as the proximal tubule (PT) and distal tubule, as well as glomeruli, podocytes, and mesangial cells [12, 19–23]. PPAR*γ* is speculated to have renoprotective effects. For example, PPAR*γ* seems to attenuate podocyte damage. Kanjanabuch et al. showed that PPAR*γ* agonists prevented podocyte injury [24]. Additionally, they showed that TZDs increased PPAR*γ* expression and activity in cultured puromycin-injured mouse podocytes [24]. Other studies have shown that although PPAR*γ* agonist treatment cannot rescue renal function, it does raise adiponectin levels in mice [25]. As adiponectin improves podocyte recovery [25], PPAR*γ*, together with adiponectin, may have some protective roles in podocytes.

The activation of PPAR*γ* by TZDs seems to protect mesangial cells from the development of diabetic change via the inhibition of inflammatory cascades [26] or TGF-*β* signaling cascades [27]. The activation of glomerular PPAR*γ* may have potential for the treatment of diabetic nephropathy. However, the detailed mechanism by which PPAR*γ* exerts its protective effects on the kidney as a whole remains to be clarified.

## 3. Thiazolidinediones (TZDs): Multipotent Roles in Glucose Metabolism

Thiazolidinediones (TZDs) were first discovered as insulin-sensitizing drugs [28]. In 1995, they were found to enact their pharmacological effects by binding to and activating PPAR*γ* [29, 30]. TZDs act as agonists for PPAR*γ* and ameliorate insulin sensitivity in the liver, muscle, and adipocytes [31–35]. There are several views on the manner in which TZDs enhance insulin sensitivity. One view is that TZDs enhance insulin signaling by stimulating insulin receptor substrate 1 (IRS-1) and inhibiting the MAPK pathway [34]. Another is that TZDs act in adipose tissue to increase adiponectin secretion while inhibiting lipolysis [2, 31] and the release of inflammatory cytokines, such as transforming growth factor-*β* (TGF-*β*). Recently Spiegelman and colleagues proposed that TZDs inhibit the phosphorylation of PPAR*γ* at Ser273 by cyclin-dependent kinase (Cdk) 5, thus preventing the development of insulin resistance [36]. They also suggested that the phosphorylation of PPAR*γ* is blocked by the inhibition of MEK/ERK. In this study, Cdk5 was shown to suppress the MEK/ERK cascade, which suggests that Cdk5 controls PPAR*γ* function [37].

In some animal models of diabetic nephropathy, such as Zucker diabetic fatty rats and Wister fatty rats, TZDs have been shown to reduce mesangial matrix volume, decrease proteinuria, and prevent the aggravation of renal function [38, 39]. TZDs have also been shown to inhibit the mRNA expression of cell matrix proteins (e.g., collagen and fibronectin) and TGF-*β* in mouse mesangial primary culture cells [27], pregnant diabetic rat models [40], and a mouse mesangial cell line [41], which indicates that TZDs inhibit mesangial cell proliferation. These results suggest that TZDs indirectly protect glomeruli against diabetic changes. Moreover, TZDs have been reported to have other renoprotective effects, such as the lowering of blood pressure, blood glucose, and insulin levels and the reduction of microalbuminuria in experimental animals, such as obese Zucker rats, streptozotocin-induced diabetic rats, and a rat model of partial nephrectomy [42, 43]. However, TZDs do not seem to reduce marcoalbuminuria in humans [43].

Also in humans, TZDs have been suggested to improve glucose homeostasis, lower blood pressure, and reduce microalbuminuria, unlike other antidiabetic drugs, such as insulin, sulfonylureas, and *α*-glucosidase inhibitors [44, 45]. Recently, TZDs were shown to prevent the onset of diabetes mellitus (DM) in persons with impaired glucose tolerance in a randomized, double-blind, and placebo-controlled clinical study [46]. On the other hand, some studies have shown that the decrease in urinary albumin-creatinine ratio after TZD treatment was comparable to that observed after gliclazide [47] and insulin [48] treatment. The above data show that treatment with TZDs can reduce microalbuminuria and may prevent the onset of DM. However, currently no studies have shown that TZDs can prevent the development and progression of human chronic kidney disease.

## 4. The Side Effects of TZDs

TZDs have many beneficial effects, including preventing the emergence and progression of DM and hypertension and their complications and preventing vicious phenomena, such as endothelial-mesenchymal transition (EMT), inflammatory responses, and fibrosis [46]. However, TZDs also have some important side effects [49]. Troglitazone has been withdrawn from the market because it was found to cause fatal liver dysfunction. Clinically, renal sodium retention and congestive heart failure (CHF) are probably the most important and troublesome side effects of TZDs. Plasma volume expansion and cardiac failure make the treatment of DM complicated [50]. Additionally, cardiovascular risks and concerns of TZDs raising mortality by causing CHF have been presented [51, 52].

TZDs also seem to increase vascular permeability in several tissues, which contributes to producing peripheral edema. Rosiglitazone was shown to enhance vascular permeability selectively in adipose tissues and retina, but not in muscle [53]. Vascular endothelial growth factor (VEGF) may be responsible for the increment of vascular permeability in the adipocytes [54].

TZDs might also cause bone fracture. Rosiglitazone is suggested to decrease bone mineral density and increase bone turnover in menopausal women; however, further investigations are required to clarify the mechanism of this effect of rosiglitazone [55–57]. At present, the most important matter of controversy regarding TZDs is probably the possibility that pioglitazone can cause bladder cancer [58, 59].

## 5. TZDs and Congestive Heart Failure (CHF)

Sodium retention accompanied with the use of TZDs sometimes makes the continuous use of TZDs difficult or impossible due to severe CHF. Approximately 5% of patients using TZDs develop peripheral edema. However, when used with other antidiabetic drugs, the risk of peripheral edema increases to approximately 18% [60]. Additionally, the risk of edema caused when 8 mg rosiglitazone is taken with insulin is 16.2%, compared to 4.7% for insulin alone [61]. However, TZDs are not thought to worsen cardiac function by themselves [62]. In the PROactive 05 study, pioglitazone treatment resulted in 28% reduction of fatal and nonfatal myocardial infarctions and 37% reduction of acute coronary syndromes compared to placebo [63]. CHF induced by TZD administration is thought to be due to renal sodium retention. At present, TZDs do not seem to increase mortality due to CHF [64]; however, there are some counterarguments regarding this point, as described above [51, 52]. According to the American Diabetes Association and the American Heart Association recommendation, patients suffering from NYHA class III or IV CHF should not take TZDs [60, 65].

## 6. The Mechanism of TZD-Induced Renal Sodium and Water Retention

As mentioned above, edema and CHF caused by TZDs are great issues clinically. In Sprague-Dawley rat models, Song et al. first showed that renal sodium retention due to an increase of tubular transporters and a decrease in glomerular filtration rate is the main cause of volume expansion by TZDs [13]. However, the detailed molecular mechanism of renal sodium retention by the kidney is still in dispute. At first, the epithelial sodium channel (ENaC) was thought to be the main cause of this volume expansion. Guan and colleagues reported that mice treated with TZDs showed weight gain which was blocked by amiloride. On the contrary, in* AQP2-Cre* x* Pparg* ^flox/flox^ mice, with selective deletion of* Pparg* from the collecting duct, TZDs did not cause volume expansion. In primary culture of IMCD cells from* AQP2-Cre* x* Pparg* ^flox/flox^ mice, pioglitazone failed to enhance amiloride-sensitive sodium transport, but it significantly enhanced amiloride-sensitive sodium transport in control IMCD cells. Additionally, as in mouse IMCD cells, pioglitazone treatment increased* Scnn1g* mRNA, suggesting that pioglitazone enhanced ENaC-*γ* subunit expression [14].

Zhang and colleagues [15] also showed that mice with collecting duct-specific knockout of the PPAR*γ* gene were resistant to TZD-induced weight gain and plasma volume expansion. In primary cultured collecting tubule cells of mice expressing PPAR*γ*, TZDs enhanced sodium transport. However, in cells lacking PPAR*γ*, TZDs did not enhance sodium transport. These two works suggest that TZDs induce plasma volume expansion by increasing sodium transport via ENaC in the cortical collecting duct (CCD). In particular, PPAR*γ* was thought to mediate the enhancement of the expression of the ENaC-*γ* subunit. Moreover, another study [66] suggested that serum glucocorticoid regulated kinase 1 (SGK1) mediates the stimulatory effect of TZDs on ENaC.

However, other studies did not support the conclusion that TZDs enhance sodium transport via the activation of ENaC in the CCD. In well-established cell lines, such as A6, M-1, and mpkCCD_cl⁡4_, insulin is known to stimulate ENaC activity. However, in these cells, TZDs failed to directly augment basal or insulin-stimulated Na^+^ flux via ENaC [18]. This clearly contradicts the view that TZDs enhance ENaC activity via PPAR*γ* regulation. Additionally, in the kidneys of Sprague-Dawley rats, TZDs failed to upregulate the expression of any ENaC subunit [13]. Vallon and colleagues showed that mice with conditionally inactivated ENaC*α* in the collecting duct showed almost the same level of fluid retention after TZD treatment as control mice. In patch clamp studies using primary cultured collecting duct cells, a nonselective cation channel, not ENaC, was activated by TZDs. They also showed that TZDs repress ENaC activity in mice, both in the acute phase (several hours) and chronic phase (days) [16, 17]. Moreover, others showed that TZDs did not enhance the ENaC promoter [17]. These results certainly argue against the view that TZDs enhance ENaC in the CCD.

Some studies have suggested that renal PT transport is stimulated by TZDs, both in animals [67] and humans [68]. Based on these observations, we speculate that TZD-induced volume expansion is multifactorial and that PT could be another target segment for TZDs. Furthermore, the “aldosterone escape” phenomenon should be considered: even if aldosterone enhances ENaC activity in the collecting duct, it suppresses sodium reabsorption in other nephron segments. Therefore ENaC activation by aldosterone excess alone does not usually induce massive volume expansion with edema formation [69].

We found [12] that TZDs markedly stimulate bicarbonate-coupled sodium transport in isolated PTs of rabbits, rats, and humans. TZDs activated both a sodium-bicarbonate cotransporter (NBCe1) and a sodium/proton exchanger (NHE3) through the PPAR*γ*/Src/EGFR/ERK pathway. However, in mice, TZDs failed to stimulate PT transport both in vivo and in vitro. This is consistent with a previous report that showed that Src/EGFR/ERK is constitutively activated in mice [70].

TZDs trigger various rapid cellular signaling events, including the activation of kinase signaling pathways, such as phosphatidylinositol 3-kinase (PI3K), Akt, ERK, and MAPK pathways, in a nongenomic manner [71]. We transfected mouse embryonic fibroblast cells from PPAR*γ* ^−/−^ mouse with the ligand binding domain of PPAR*γ*. This experiment confirmed the presence of nongenomic signaling that resulted in the activation of ERK; this signal required PPAR*γ* to have ligand-binding ability but did not require the transcription of PPAR*γ* [12]. Additionally we showed that TZDs rapidly facilitate the association of PPAR*γ* with Src, which is also dependent on the ligand-binding ability of PPAR*γ*. These results, together with the rapid kinetics of responses that are independent of transcriptional activity, indicate that PPAR*γ* can activate the ERK pathway through nongenomic mechanism, similar to another nuclear receptor, estrogen [72]. The dependence on Src, the association between PPAR*γ* and Src, and the negative effect of constitutive Src activation in PPAR*γ*-dependent nongenomic signaling support the central role of Src in this signaling pathway. The magnitude of the enhancement of PT transport by TZDs is comparable to, or even exceeds, that of angiotensin II [73]. In PT, angiotensin II is thought to be the strongest stimulatory hormone. Therefore, we concluded that the stimulation of renal PT transport via PPAR*γ*-dependent, nongenomic signaling may play an important role in the plasma volume expansion induced by TZDs [12].

Other channels/transporters have also been suggested to be regulated by PPAR*γ* and its agonists, TZDs. The expression level of aquaporin 3 (AQP3) mRNA in the renal outer medulla was stronger in TZD-treated Otsuka Long-Evans Tokushima Fatty (OLETF) rats than in OLETF rats without TZD treatment and control LETO rats [74]. Another study showed that TZD treatment increased the expression of AQP3 protein in diabetic* db/db* mice but not in wild-type mice. Another aquaporin, AQP2, was downregulated in lean wild-type mice but not in* db/db* mice [75]. Aquaporins have 13 subtypes, and many of them are expressed widely in nephron segments and are mainly involved in water transport [76, 77]. In particular, AQP2 is located in CCD and is known as a target for vasopressin [78]. AQP3 is located in the basolateral side of the collecting duct and is involved in water reabsorption [79]. Additionally, in the kidney of Sprague-Dawley rats, the protein expression of NHE3 and NKCC2 was elevated after TZD treatment [13]. NKCC2 reabsorbs sodium and potassium coupled with chloride, predominantly in the apical side of the thick ascending limb of Henle (TAL) [80, 81]. These results strongly suggest that the volume expanding effect of TZDs is multifactorial. Recently Fu and colleagues have reported that ENaC in the connecting tubule may play a role in the fluid retention induced by TZD [82].  Table 1 summarizes the potential targets of TZDs in the PT and TAL. The controversial data as to the potential effects of TZDs on ENaC are summarized in Table 2.

The development of new TZDs with less side effects seems to be difficult. Rivoglitazone was not released because its effects and side effects were not significantly different from those of pioglitazone [83]. A selective PPAR*γ* activator with less frequency of edema, INT131, is now being examined by a clinical trial [84]. Such drugs may help in the therapy of DM in the near future.

## 7. Conclusions

We have overviewed PPAR*γ*, its agonists, TZDs, and their side effects with a focus on the mechanisms of edema and sodium retention. TZDs are highly effective antidiabetic drugs with unique functions, such as a renoprotective effect, amelioration of glucose homeostasis, and blood pressure lowering, that other antidiabetic drugs do not have. However, the use of TZDs is often associated with edema and CHF, which make it impossible to use TZDs in case of severe CHF. The mechanism by which TZDs induce volume expansion may be multifactorial, as shown in Table 1. At first ENaC in the CCD was thought to play a central role in TZD-induced volume expansion; however, the results of other studies have not supported this view and suggested the involvement of other transporters in the CCD. We have found that NBCe1 and/or NHE3 in the PT may play a significant role in TZD-induced sodium retention through a PPAR*γ*-dependent nongenomic mechanism. Other sodium and water transporters, such as NKCC2, AQP2, and AQP3, have also been proposed as targets for TZDs. The development of novel TZDs or PPAR*γ* modulators with less side effects is expected.

## Figures and Tables

**Table 1:** The proposed targets and effects of TZDs in PT and TAL.

Nephron segment	Targets	Species	Materials	Effects	Citation
PT	NBCe1	Rat Rabbit Human	Isolated proximal tubule	Stimulation of transport	[12]
PT	NHE3	Rabbit	Isolated proximal tubule	Stimulation of activity	[12]
PT	NHE3	Sprague-Dawley rat	Total kidney homogenate	Enhancement of protein expression	[13]
TAL	NKCC2	Sprague-Dawley rat	Total kidney homogenate	Enhancement of protein expression	[13]

**Table 2:** Different effects of TZDs on ENaC.

Nephron segment	Species	Materials	Effects	Citation
Collecting duct	Mouse	Primary cultured IMCD cells	Upregulation of ENaC-*γ* mRNA expression	[14]
Collecting duct	Mouse	Primary cultured CD cells	Increased Na transport (suppressed in CD PPAR KO)	[15]
CCD	Mouse	Split-opened isolated CCD	Channel activity not altered	[16]
Cortex	Mouse	Kidney cortex lysate	Decrease in ENaC-*α* and -*β* subunit mRNA expression Decrease in ENaC-*γ* subunit protein expression	[17]
CCD	Mouse	M1 cell line	Decrease in ENaC-*α* and -*γ* subunit mRNA expression	[17]
CCD	Mouse	M1 cell line mpkCCD_cl4_ cell line	No direct enhancement of Na^+^ flux via ENaC	[18]
Kidney	*Xenopus laevis *	A6 cell line	No direct enhancement of Na^+^ flux via ENaC	[18]
